# Comparison of the Efficacy of Danhong Injections at Different Time-points During the Perioperative Period of Acute Myocardial Infarction: A Systematic Review and Meta-analysis of Randomized Controlled Trials

**DOI:** 10.3389/fphar.2021.643446

**Published:** 2021-04-29

**Authors:** Qing-Ying He, Xin-Yu Yu, Zheng Xiao, Xin Sun, Wei-Feng Zhu, Xing-Qian Yi, Qian Chen, Jia-Hui Zhang, Shu-Xian Chen, Xu Zhou, He-Yun Nie, Hong-Cai Shang, Xiao-Fan Chen

**Affiliations:** ^1^ Evidence-Based Medicine Research Centre, Jiangxi University of Traditional Chinese Medicine, Nanchang, China; ^2^ Preventive Treatment Center, Hongdu Hospital of Traditional Chinese Medicine Affiliated to Jiangxi University of Traditional Chinese Medicine, Nanchang, China; ^3^ Department of General Practice, Affiliated Hospital of Zunyi Medical University, Zunyi, China; ^4^ Chinese Evidence-based Medicine Centre and CREAT Group, State Key Laboratory of Biotherapy, West China Hospital, Sichuan University, Chengdu, China; ^5^ Key Laboratory of Chinese Internal Medicine of Ministry of Education and Beijing, Dongzhimen Hospital, Beijing University of Chinese Medicine, Beijing, China

**Keywords:** danhong injection, acute myocardial infarction, intervention time point, systematic review, meta-analysis

## Abstract

**Objectives:** Danhong injections (DHI) are widely used in the treatment of acute myocardial infarction (AMI). As there are no guidelines for the timing of DHI in the peri-percutaneous coronary intervention (PCI) period for AMI, we investigated the effects of DHI timing.

**Methods:** We reviewed reports published before September 30, 2020 in PubMed, embase, the Cochrane Central Register of Controlled Trials, the Chinese BioMedical database, Chinese VIP database, Wanfang database, and Chinese National Knowledge Infrastructure database. Only randomized controlled trials of DHI with percutaneous coronary intervention for AMI were included. Methodological quality was assessed using the Cochrane evaluation manual 5.3.3 criteria. A meta-analysis was performed, and forest plots were drawn.

**Results:** We included 23 studies which all revealed that patients in DHI groups had better efficacy than control groups. Subgroup analysis revealed that DHI administered intraoperatively and continued postoperatively was more effective in increasing left ventricular ejection fraction when compared to other time-points (*p* < 0.001). The pre- and intraoperative use of DHI could improve reflow more effectively than conventional treatment, while the effect was not significant in the postoperative intervention study (*p* = 0.654). The 16 postoperative interventions revealed that the effect of DHI at 14 days was better than that at 7 and 10 days for hs-CRP (*p* = 0.013), the 10-days treatment produced better results for CK-MB than for the other treatments (*p* < 0.001) and a dosage of 30 ml proved most effective for IL-6 (*p* < 0.001).

**Conclusion:** DHI proved to be superior to conventional Western medicine in reducing the incidence of adverse cardiac events, promoting reperfusion, improving cardiac function, reducing inflammatory factors, and protecting the myocardium. DHI should be administered early in the perioperative period and continued postoperatively because of its ability to improve cardiac function. Furthermore, in the PCI postoperative, 30 ml is recommended to inhibit IL-6 levels, for patients with high hs-CRP, a course of 14 days is most effective, for patients with obvious abnormalities of CK-MB, a 10-days course of treatment is recommended. However, due to the limited number and quality of the original randomized controlled trials, our conclusions need large, multi-centre RCTs to validation.

## Introduction

Acute myocardial infarction (AMI) is a common cardiovascular disease with high morbidity and mortality, which is mainly caused by plaque rupture that triggers a thrombus and thereby blocks the coronary artery (Ma et al., 2020; Virani et al., 2020). This coronary occlusion causes interruption of blood flow, and severe sustained ischemia leads to the development of partial myocardial necrosis, shock, or heart failure. Currently, percutaneous coronary intervention (PCI) is the first choice of treatment for AMI (Brown and Gupta, 2020). A number of studies have shown that occluded blood vessels could be restored by PCI to reduce AMI mortality (Ozaki et al., 2018). However, during PCI, some ruptured thrombus detachments could follow the blood flow to the distal microvasculature, which could easily cause non-reperfusion or slow flow in the coronary arteries, resulting in coronary non-perfusion and local myocardial ischemia (Cho et al., 2017; Zeitouni et al., 2018). Previous studies have found that a high thrombotic burden in patients with myocardial infarction is independently associated with distal embolism, non-reperfusion, myocardial necrosis, and major cardiac adverse events (MACEs) (Brener et al., 2014; Napodano et al., 2014; Costa et al., 2015). Thus, it is necessary to administer antithrombotic therapy in the perioperative period, which mainly consists of antiplatelet and anticoagulant therapy.

In the European Society of Cardiology guidelines, a series of pre-, intra-, and postoperative treatments are recommended. Preoperatively for antiplatelet aggregation, a loading dose of aspirin of 150–300 mg orally (class of recommendation: Ⅰ; level of evidence: B) and P2Y_12_: prasugrel 60 mg orally, ticagrelor 180 mg orally, or clopidogrel 600 mg orally (class of recommendation: Ⅰ; level of evidence: A) are recommended (Dickstein et al., 2008). Preoperatively for intraoperative anticoagulation, unfractionated heparin (class of recommendation: Ⅰ; level of evidence: C), enoxaparin (class of recommendation: Ⅱa; level of evidence: A), and bivalirudin (class of recommendation: Ⅱa; level of evidence: A) are recommended (Swedberg et al., 2005). After surgery, dual antiplatelet therapy (routine dose of aspirin and P2Y_12_) is recommended (class of recommendation: Ⅰ; level of evidence: A) (Dickstein et al., 2008). Furthermore, when there is a separate indication for full-dose anticoagulation, for instance, due to atrial fibrillation, mechanical valves, or left ventricular (LV) thrombus, or prophylactic doses for the prevention of venous thromboembolism in patients requiring prolonged bed rest, routine post-procedural anticoagulant therapy is needed (class of recommendation: Ⅱa; level of evidence: A) (Ibánez et al., 2017).

**FIGURE 1:**
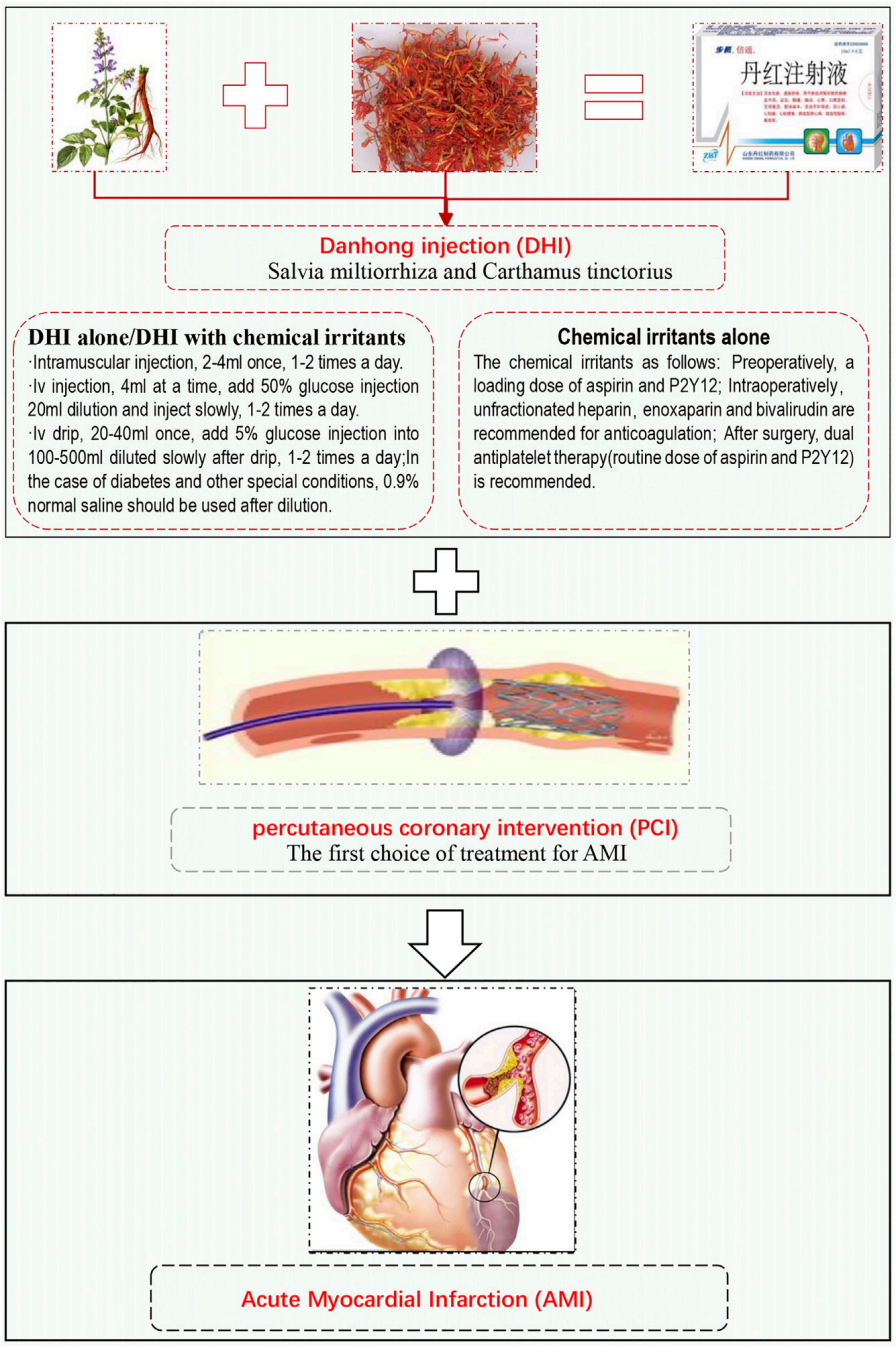
Danhong injection for perioperative period of acute myocardial infarction.

Danhong injection (DHI) is a traditional Chinese medicine registered in the State Food and Drug Administration of China, which is prepared by combining active ingredients from Salvia miltiorrhiza Bunge [Lamiaceae] and Carthamus tinctorius L[Asteraceae] with excipients that include sodium hydroxide and water (Zou et al., 2018) (Supplementary Figures S1–S3; Table S1). The protective mechanisms of DHI include inhibition of oxidative stress and inflammation, anticoagulant and antithrombotic effects, reduction of apoptosis, reduction of blood pressure, relaxation of blood vessels, and promotion of angiogenesis (Feng et al., 2019). In China, DHI is widely used in the prevention and treatment of myocardial infarction, which is recommended by many current Chinese guidelines for the perioperative period of AMI (Chen et al., 2014; Doctor Society of Integrative Medicine, 2018; Fu et al., 2018). However, these guidelines and consensus do not address the specific timing of DHI administration during the perioperative period, and no current systematic evaluation of timing for DHI has been reported. Therefore, based on the pre-, intra-, and postoperative intervention strategies mentioned in the current guidelines, we performed a systematic review and meta-analysis to summarize the current evidence from randomized controlled trials (RCTs) on the efficacy of DHI for the treatment of AHI at different peri-PCI stages (Figure 1).

## Materials and Methods

The study was conducted in accordance with the Preferred Reporting Items for Systematic Reviews and Meta-Analyses (PRISMA) guidelines.

### Data Source and Search Strategy

Literature was retrieved from the following databases: PubMed, embase, the Cochrane Central Register of Controlled Trials and four Chinese databases (WanFang Medical database, Chinese BioMedical database, Chinese VIP database, and China National Knowledge Infrastructure). The retrieval dates were from their respective inception dates to September 30, 2020. In order to screen the potential eligible studies, entries related to AMI, PCI, and DHI were searched. No language or publication status restrictions were set. Search strategies are listed in Supplementary Table S2, and the search terms were modified in different databases. We also searched the reference lists of review articles and identified any possible matching RCTs.

### Eligibility Criteria

#### Types of Participants

The inclusion criteria for patients were as follows: Patients who were confirmed to have had AMI and were in the perioperative period of PCI, regardless of whether the diagnostic criteria followed the Chinese or non-Chinese guidelines. The perioperative period was defined as the whole process around the operation, from the decision of the patient to receive surgical treatment to the basic recovery, including the period of time before, during, and after PCI.

#### Types of Interventions

The study population was divided into a control group, i.e., patients with AMI who received conventional treatment, such as antiplatelets and anticoagulants, during the peri-PCI period, and an observation group, in which the patients received either DHI alone or DHI in combination with conventional treatment. There was no restriction on dosage, administration route, or follow-up time for either intervention measures during the peri-PCI period.

#### Types of Outcome Measures

Reducing the incidence of postoperative cardiovascular events is the primary goal of PCI treatment. Reperfusion after PCI directly affects the incidence of MACEs. Therefore, MACEs and reperfusion were selected as the primary outcome. Secondary observation outcomes contained levels of cardiac function, myocardial injury, and inflammatory factor. The evaluation index of myocardial injury: the guidelines recommend cardiac troponin T (cTnT) as the preferred indicator and creatine kinase (CK)-MB as the alternative indicator, inflammatory factor indicators hypersensitive C-reactive protein (hs-CRP) and interleukin (IL)-6. LV ejection fraction (LVEF) was used to evaluate cardiac function.

#### Types of Studies

RCTs with complete information, based on blinding or not, with no limit on time, language, and publication type, were included.

### Exclusion Criteria

Studies with the following characteristics were excluded: 1) those in which the study subjects had angina pectoris, heart failure, or other coronary heart disease; 2) those the time-points of perioperative PCI is not indicated; 3) those interventions were combined with other TCM interventions (such as acupuncture, proprietary Chinese medicine, etc.) 4) those without corresponding outcome indicators; 5) those in which the study type was a non-randomized trial, cohort study, or a case‒control study; 6) those obvious flaws, such as data duplication or statistical errors; and 7) Those articles that only have abstracts and are not available in full.

### Data Extraction and Quality Assessment

All papers were screened by two researchers (XY and QC) as per the inclusion and exclusion criteria. The following data were extracted: 1) characteristics of the study: authors, year, study design, sample size; 2) characteristics of patients: age, sex, basic diseases, myocardial infarction site, etc; 3) intervention: trial group intervention, control group intervention, drug intervention time, dose; and 4) outcomes of the study, such as cardiac function indicators, inflammation levels, and myocardial injury. Two researchers cross-checked the data extracted from the selected literature. Differences were resolved by discussion with a third researcher (QH).

The Cochrane evaluation manual 5.3.3 was used as a benchmark to evaluate the quality of the studies, based on the following aspects: Random serial generation; allocation scheme hiding; blinding method (patients, medical staff, outcome evaluations, and data analysis); data integrity (follow-up rate and important indicators); selective reporting; and other sources of bias (such as baseline imbalance, suspected fraud, etc.). According to the above indicators, the two researchers’ answer of “yes” indicated a lower risk of bias, “no” indicated a higher risk of bias, and “unclear” indicated an uncertain risk of bias.

### Data Synthesis

Stata v 16.0 software was used to perform meta-analysis on subjects (StataCorp, College Station, TX, United States of America). Count data are expressed as relative risk (RR) and 95% confidence intervals (CIs), and continuous variables are presented as the standardized mean diﬀerence (SMD) with 95% CIs. Heterogeneity was analyzed using the chi-square test. If the results of showed statistical homogeneity (*p* > 0.1, I^2^ ≤ 50%), fixed-effect models were used for meta-analysis, whereas if the results showed statistical heterogeneity (*p* < 0.1, I^2^ > 50%), random-effects models were adopted. The source of heterogeneity was analyzed, and subgroup analysis, sensitivity analysis, and meta regression were applied to analyze heterogeneity between the study results.

## Results

### Literature Search Results

**FIGURE 2:**
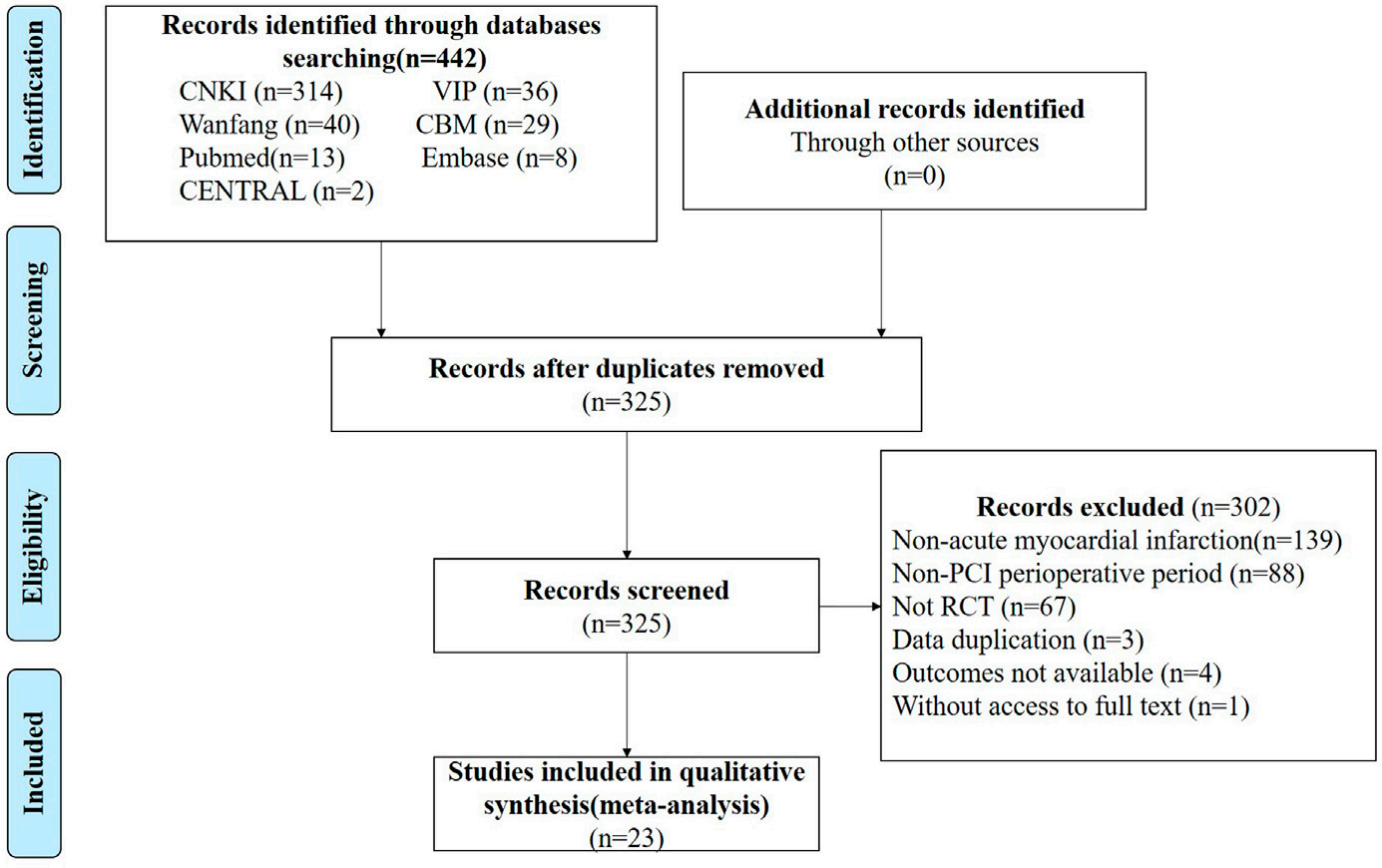
Process of study extracted for the meta-analysis.

The retrieval flow chart shown in Figure 2 describes the literature search process and study selection. After duplicates were removed, 325 articles were identified and read. According to the PICOS principle, we excluded 139 studies that did not involve AMI, 88 that administered thrombolytic therapy rather than PCI, 67 non-RCTs, three studies with significant data duplication, four with inconsistencies in observational indicators, and one study with no full text available. Finally, 23 RCTs were included.

### Study Characteristics

#### Diagnostic Criteria and Intervention Time

The 23 Chinese studies involved 2,639 patients, with 1,333 in the experimental groups and 1,306 in the control groups. The age of patients ranged from 41 to 79 years, and baseline data of age, sex, basic disease had no difference between the two groups as shown in Table 1. Three of these studies used DHI before surgery, three used it intraoperatively, one used it both in intra- and post-operatively, and 16 used it after PCI. In terms of the diagnosis of AMI, nine articles (Chen et al., 2010; Lv, 2012; Cui and Wang, 2014; Xu et al., 2015; Zheng et al., 2015; Liu et al., 2017; Zeng et al., 2017; Hu, 2020; Li and Xu., 2020) were based on the guidelines of the Chinese Society of Cardiology (Gao, 2001; Medicine, 2009; Members, 2015), five (Gao et al., 2008; Qin et al., 2014; Jia et al., 2015; Leng, 2016; Qin et al., 2018) on the guidelines of the World Health Organization or the American Heart Association (JM, 1979; Antman et al., 2004), three (Huo et al., 2019; You, 2019; Lv, 2020) on the third universal definition of myocardial infarction (Thygesen et al., 2012), and six did not specify the diagnostic criteria (Wang B. et al., 2013; Wang et al., 2015; Huang, 2016; Li and Meg, 2017; Wu et al., 2017; Li et al., 2019).

**TABLE 1:** Other characteristics of included studies.

Study (author/year)	Sample size (T/C)	Hypertension (T/C)	Diabetes mellitus (T/C)	Dyslipidemias (T/C)	Smoke (T/C)	Drink (T/C)	BMI (T/C)	Killip scale (T/C)	Site of myocardial infarction (T/C)
Huang (2016)	100 (50/50)	NR	NR	NR	NR	NR	NR	NR	NR
Leng (2016)	240 (120/120)	43/41	29/32	NR	NR	NR	NR	NR	AWMI: IWMI: LWMI (74:18:28)/(76:20:24)
Jia et al. (2015)	120 (60/60)	20/22	25/24	NR	27/28	NR	(28.06 ± 4.26)/(27.54 ± 4.91)	NR	NR
Qin et al. (2018)	126 (63/63)	NR	NR	NR	NR	NR	NR	NR	NR
Wu et al. (2017)	80 (44/36)	NR	NR	No details	NR	NR	No details	No details	NR
Wang (2013)	60 (30/30)	20/20	22/21	11/12	22/21	NR	NR	10/10 (>Ⅱ level)	NR
Li and Xu (2020)	166 (86/80)	NR	NR	NR	NR	NR	NR	Ⅱ: Ⅲ: Ⅳ (28:48:10)/(25:46:9)	AWMI: IWMI: LWMI (36:24:26)/(32:22:26)
Lv (2020)	100 (50/50)	28/26	21/23	NR	NR	NR	NR	10/11(>Ⅰ level)	NR
Hu (2020)	86 (43/43)	NR	13/16	23/19	20/23	21/25	NR	NR	AWMI: IWMI: LWMI: RVMI (15:8:15:5)/(16:7:14:6)
You (2019)	119 (62/57)	32/24	16/13	16/23	29/37	NR	(25.8 ± 3.8)/(25.9 ± 4.3)	NR	NR
Li et al. (2019)	90 (45/45)	NR	NR	NR	NR	NR	NR	NR	NR
Huo et al. (2019)	80 (40/40)	NR	NR	NR	NR	NR	NR	NR	NR
Liu et al. (2017)	180 (90/90)	NR	NR	NR	NR	NR	NR	NR	NR
Li et al. (2017)	85 (44/41)	24/20	11/10	33/23	24/21	NR	NR	NR	NR
Zeng et al. (2017)	120 (60/60)	28/26	14/15	12/14	23/20	NR	NR	NR	NR
Zheng et al. (2015)	300 (150/150)	NR	NR	NR	NR	NR	NR	NR	NR
Xu et al. (2015)	71 (36/35)	25/26	9/10	NR	NR	NR	NR	8/6 (>Ⅰ level)	AWMI: IWMI: LWMI (17:15:4)/(16:13:6)
Wang et al. (2015)	64 (34/30)	12/10	6/4	5/5	10/8	NR	NR	NR	NR
Qin et al. (2014)	112 (56/56)	26/25	10/11	NR	30/32	NR	NR	Ⅰ: Ⅱ-Ⅲ: Ⅳ (44:8:4)/(43:1:3)	AWMI: IWMI: LWMI (29:19:8)/(19:18:7)
Cui and Wang (2014)	180 (90/90)	NR	NR	NR	NR	NR	NR	NR	NR
Lv (2012)	40 (20/20)	15/15	7/5	NR	11/12	NR	NR	NR	NR
Chen et al. (2010)	59 (29/30)	NR	NR	NR	NR	NR	NR	NR	AWMI: IWMI: IWMI + LWMI: IWMI + RVMI: IWMI + RVMI + LWMI (16:5:3:3:2)/(17:5:4:2:2)
Gao et al. (2008)	61 (31/30)	21/20	13/14	NR	16/15	NR	(25.8 ± 3.5)/(25.8 ± 3.2)	(1.4 ± 0.6)/(1.4 ± 0.8)	AWMI: IWMI: AWMI + IWMI (15:9:7)/(3/11/6)

NOTE: NR, no report; AWMI, Anterior wall myocardial infarction; IWMI, Inferior wall myocardial infarction; LWMI, Lateral wall myocardial infarction; RVMI, Right ventricular myocardial infarction.

#### Specific Interventions

As per the treatment guidelines, routine periprocedural pharmacotherapy was followed. Antiplatelet aggregation: a loading dose of aspirin and a potent P2Y_12_ inhibitor (prasugrel, ticagrelor, or clopidogrel) were recommended before (or at the latest at the time of) PCI. Intraoperatively, anticoagulant therapy: fractionated heparin (UFH) was recommended. Postoperatively, routine aspirin, P2Y_12,_ and UFH were recommended for the prevention of venous thromboembolism in patients requiring prolonged bed rest, at the same time, isosorbide nitrate sustained-release capsule were used to dilate coronary arteries, atorvastatin calcium tablets were used to stabilize plaques.

In terms of interventions, all three preoperative intervention studies used nitro-glycerine (200 μg) plus DHI (20 mg) compared to nitro-glycerine (200 μg) alone in the control group; in two studies, nitro-glycerine was used preoperatively, and in one study, it was added intraoperatively. The three intraoperative intervention studies used PCI plus DHI vs. PCI alone; DHI involved the injection of 4 ml of the agent intravenously immediately at the start of PCI and maintenance of a 20 ml intravenous drip until the end of the procedure. In the combined intraoperative and postoperative study, all patients received 4 ml of DHI intravenously immediately at the start of PCI and 20 ml via an intravenous drip until the end of the procedure. The observation group continued to receive 4 ml of DHI intravenously every day after the operation for 14 days. Among the 16 postoperative intervention studies, all involved DHI plus routine treatments compared with routine treatments alone, the dosage of DHI ranged from 20–40 ml, and the course of treatment ranged from 7–14 days, as shown in Table 2. The actual routine treatments used in each study differed, however, all of them are based on antiplatelet and anticoagulant therapy.

**TABLE 2:** Summary of the randomized controls trials of Danhong Injection for PMS.

Study (author/year)	Sample size (T/C)	Age (years)		Sex (Male/female)		Drug intervention time	Dosing time	Intervention		Main outcomes
		T	C	T	C			T	C	
Huang (2016)	100 (50/50)	52.13 ± 8.59	53.61 ± 9.47	32/18	31/19	Preoperative	1 day	DHI(20 mg,qd), plus(B)	(B) Nitroglycerin (200 μg)	④⑦
Leng (2016)	240 (120/120)	56.9 ± 10.7	58.1 ± 11.2	80/40	77/43	Preoperative	1 day	DHI(20 mg,qd), plus(B)	(B) Nitroglycerin (200 μg)	②⑦
Jia et al. (2015)	120 (60/60)	62.23 ± 11.26	64.56 ± 12.85	35/25	40/20	Preoperative	1 day	DHI(20 mg,qd), plus (B)	(B) nitroglycerin (200 μg)	②⑦
Qin et al. (2018)	126 (63/63)	63.98 ± 1.25	63.41 ± 1.16	29/34	33/30	Intraoperative	1 day	DHI(4 ml + 20 ml), plus(B)	(B) direct PCI+	①②④⑧
Wu et al. (2017)	80 (44/36)	NR	NR	NR	NR	Intraoperative	1 day	DHI(4 ml + 20 ml), plus(B)	(B) direct PCI	③⑦⑧
Wang D. et al. (2013)	60 (30/30)	65.22 ± 7.54	63.61 ± 8.21	23/7	21/9	Intraoperative	1 day	DHI (4 ml + 20 ml),plus(B)	(B) direct PCI	③⑧
Li and Xu (2020)	166 (86/80)	59.25 ± 6.69	58.75 ± 5.68	45/41	40/40	Intraoperative Postoperative	1 day	(B_i_) DHI (4 ml + 20 ml)	(B_i_) DHI (4 ml + 20 ml)	④
							14 days	DHI(4 ml,qd), plus (B_p_)	(B_p_) DAAT + UFH	
Lv (2020)	100 (50/50)	60 ± 5.8	59 ± 6	32/18	30/20	Postoperative	14 days	DHI(40 ml,qd), plus(B)	(B) DAAT + PS	①③⑤⑥⑦
Hu (2020)	86 (43/43)	50.28 ± 0.43	50.62 ± 0.53	28/15	27/16	Postoperative	14 days	DHI(30 ml,qd), plus(B)	(B) DAAT + CE + UFH + PS	⑦⑧
You (2019)	119 (62/57)	58.1 ± 9.9	58.0 ± 9.7	53/9	50/7	Postoperative	7 days	DHI(40 ml,qd), plus(B)	(B) DAAT	①②④⑤⑥
Li et al. (2019)	90 (45/45)	60.14 ± 4.81	60.17 ± 2.85	20/25	23/22	Postoperative	7 days	DHI(30 ml,qd), plus(B)	(B) DAAT + PS	④
Huo et al. (2019)	80 (40/40)	62 ± 4	61 ± 6	28/12	26/14	Postoperative	14 days	DHI(40 ml,qd), plus(B)	(B) DAAT + UFH + PS + CE	①③④⑤⑦
Liu et al. (2017)	180 (90/90)	NR	NR	NR	NR	Postoperative	14 days	DHI(20 ml,qd), plus(B)	(B) DAAT + UFH	④⑦
Li and Meng (2017)	85 (44/41)	65.33 ± 8.21	64.25 ± 7.58	35/9	32/8	Postoperative	14 day	DHI(40 ml,qd), plus(B)	(B) DAAT + UFH + PS	①④
Zeng et al. (2017)	120 (60/60)	65.13 ± 2.38	64.38 ± 2.12	38/22	36/24	Postoperative	14 days	DHI(40 ml,qd), plus(B)	(B) DAAT + UFH + CE	①④⑧
Zheng et al. (2015)	300 (150/150)	61.7 ± 7.4 (no details)		186/114 (no details)		Postoperative	10days	DHI(30 ml,qd), plus(B)	(B) DAAT + UFH	④⑧
Xu et al. (2015)	71 (36/35)	65 ± 13	63 ± 11	26/10	23/12	Postoperative	14 days	DHI(40 ml,qd), plus(B)	(B) DAAT + PS	①③④⑤⑥
Wang et al. (2015)	64 (34/30)	61.43 ± 7.61	60.51 ± 8.35	25/9	22/8	Postoperative	14 days	DHI(40 ml,qd), plus(B)	(B) DAAT + UFH + PS	①④
Qin et al. (2014)	112 (56/56)	52.31 ± 11.24	55.12 ± 10.52	31/25	30/26	Postoperative	7 days	DHI(40 ml,qd), plus (B)	(B) DAAT + UFH + PS	①④⑤⑥⑦
Cui and Wang (2014)	180 (90/90)	72.1 ± 6.5	72.3 ± 5.8	52/38	54/36	Postoperative	10 days	DHI(30 ml,qd), plus(B)	(B) CE + UFH + PS	⑦
Lv (2012)	40 (20/20)	67.05 ± 7.52	64.05 ± 11.46	11/9	13/7	Postoperative	10 days	DHI(20 ml,qd), plus(B)	(B) CT	④⑥⑧
Chen et al. (2010)	59 (29/30)	61.9士5.2	65.2 ± 4.5	22/7	21/9	Postoperative	14 days	DHI(20 ml,qd), plus (B)	(B) DAAT + UFH	③④
Gao et al. (2008)	61 (31/30)	60.1士10.6	59.8士7.6	20/11	18/12	Postoperative	14 days	DHI(40 ml,qd), plus(B)	(B) DAAT + CE + UFH	①⑦

Note: T, trial group; C, control group; NR, no report; CT, conventional therapy, no details were given; DAAT, Double antiplatelet aggregation (clopidogrel bisulfate + aspirin enteric tablets); UFH, fractionated heparin; CE, coronary enlargement (Isosorbide nitrate sustained-release capsule); PS, plaque stabilization (atorvastatin Calcium Tablets). ①MACEs; ② Reflow; ③STR; ④LVEF; ⑤cTnT; ⑥CK-MB; ⑦hs-CRP; ⑧IL-6.

#### Measurement Indicators

In terms of the observational indicators, the three preoperative intervention studies all assessed the levels of hs-CRP, two focused on reperfusion, and only one measured LVEF. In the three intraoperative intervention studies, both IL-6 and reperfusion indicators were measured. In the combination trial, only LVEF has been measured. In the 16 postoperative intervention studies, 13 studies used LVEF as the main indicator, nine focused on MACEs, seven measured hs-CRP levels, and five relied on reperfusion conditions. Five of these postoperative studies focused on CK-MB and cTnT; indicators not mentioned in the pre- and intraoperative reports. Detailed observations of the indicators reported in each study are shown in Table 2 **.**

### Risk of Bias

**FIGURE 3:**
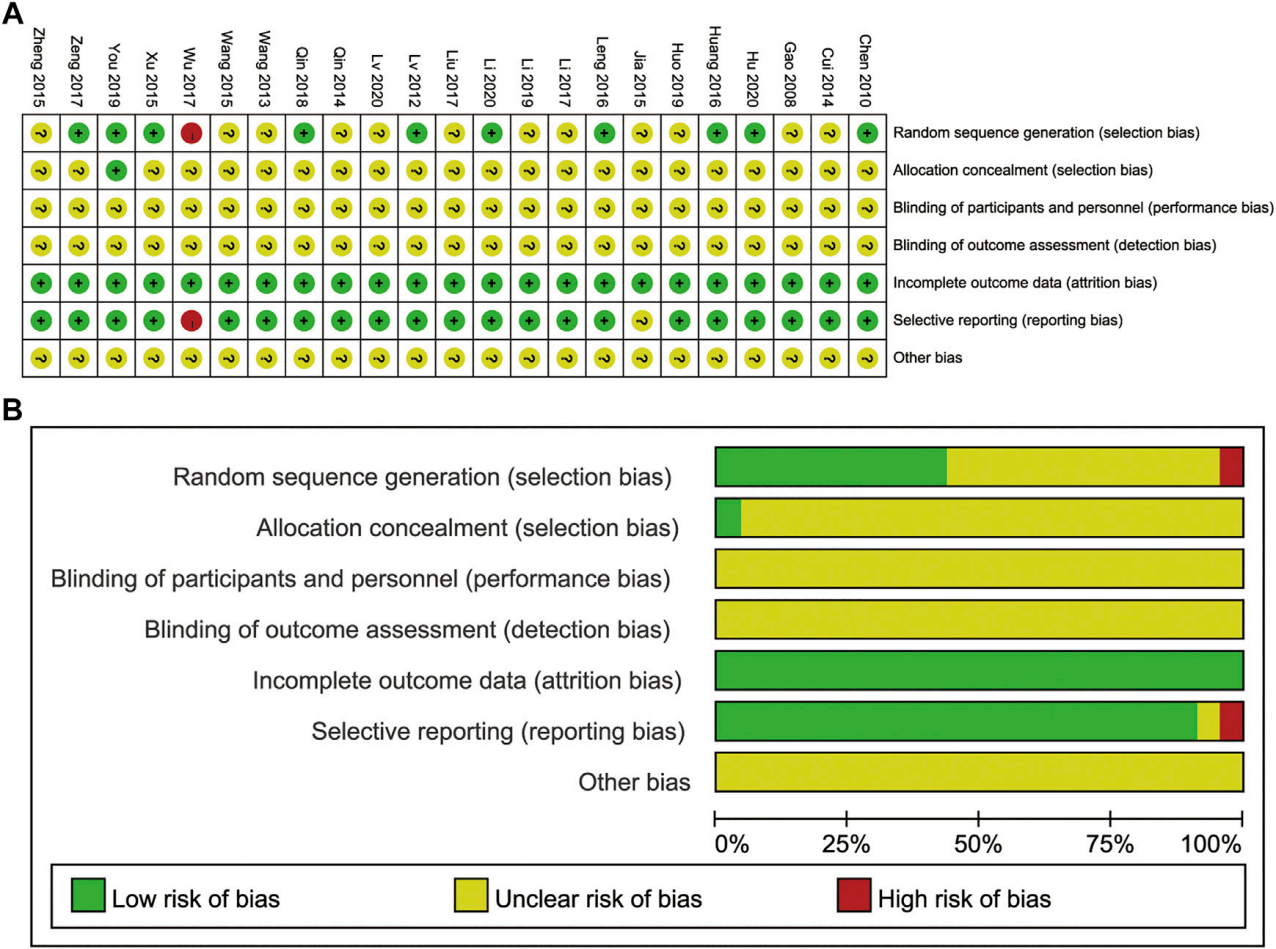
**(A)** Risk of bias summary. **(B)** Risk of bias graph.

The results for the assessment of bias risk are shown in Figure 3. We rated 10 studies as having a low risk of bias due to their implementation of a random number table or a stratified block group randomization (Chen et al., 2010; Lv, 2012; Xu et al., 2015; Huang, 2016; Leng, 2016; Zeng et al., 2017; Qin et al., 2018; You, 2019; Hu, 2020; Li and Xu., 2020). One study that used grouping by time of admission was classified as having a high risk of bias (Wu et al., 2017), while other studies only reported “randomization”. One study used a central randomization method to implement allocation hiding (You, 2019), while the remaining studies did not report the allocation concealment process, blinding of patients or personnel, or of outcome assessment. All trials involved complete follow-up. We judged one study to be at a high risk of selective reporting bias (Wu et al., 2017), because it failed to report the observation indices accurately, with underreporting. One study did not specify the outcome measures in advance (Jia et al., 2015), and thus, we judged that the risk of selective reporting was uncertain. All studies reported consistent baselines, but it was uncertain whether there were other biases.

### Outcome Measures With Subgroup Analysis

#### Incidence of MACEs

**FIGURE 4:**
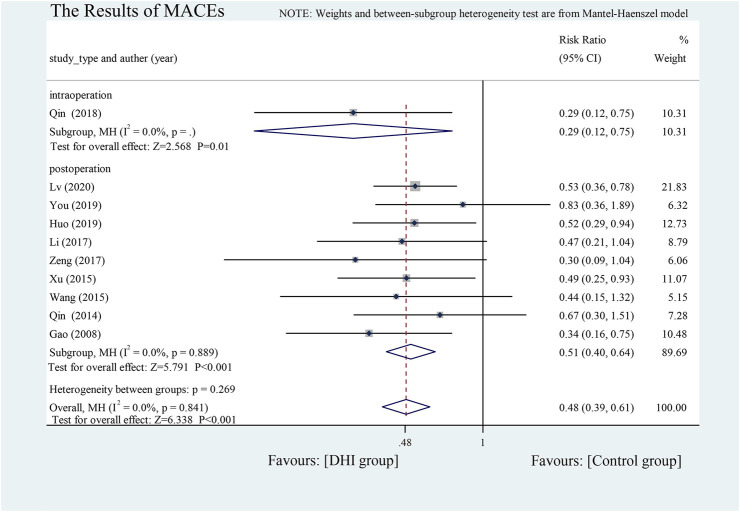
The results of MACEs.

There were 10 studies (one intra- and nine post-operative) that reported the incidence of MACEs (Gao et al., 2008; Qin et al., 2014; Wang et al., 2015; Xu et al., 2015; Li et al., 2017; Zeng et al., 2017; Qin et al., 2018; Huo et al., 2019; You, 2019; Lv, 2020). These included severe arrhythmias, angina, recurrent myocardial infarction, heart failure, and cardiogenic death. There was no significant heterogeneity among the results, so fixed-effects model was used. Overall, the incidence rate of MACEs was lower in the DHI groups (RR = 0.48, 95% CI [0.39 to 0.61], *p* < 0.001) and there were no difference between intra- and post-operative groups (*p* = 0.269) **(** Figure 4 **)**.

#### Reperfusion Conditions

Two indicators were adopted to assess the reperfusion condition: Thrombolysis in Myocardial Infarction (TIMI) flow grade and ST-segment resolution (STR) revealed on electrocardiogram. A TIMI ≥ grade 3 and an STR rate ≥50% were considered as good reperfusion (Horszczaruk et al., 2014).

**FIGURE 5:**
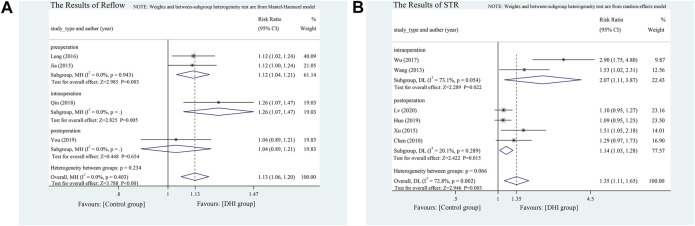
**(A)** The results of reflow. **(B)** The results of STR.

A fixed effects model was adopted and four studies found good reperfusion in the DHI group (RR = 1.13, 95% CI [1.06 to 1.20], *p* < 0.001) (Jia et al., 2015; Leng, 2016; Qin et al., 2018; You, 2019). Subgroup analysis was conducted according to different DHI intervention times, and meta-analysis revealed that the use of DHI could improve TIMI blood flow grading more effectively than conventional treatment both pre- (RR = 1.12, 95% CI [1.04 to 1.21], *p* < 0.001) and intra-operatively (RR = 1.26, 95% CI [1.07 to 1.47], *p* < 0.001). Postoperative intervention studies showed no significant differences between the treatment groups (*p* = 0.654) **(** Figure 5A **)**.

Six studies used STR to assess reperfusion (Chen et al., 2010; Wang D. et al., 2013; Xu et al., 2015; Wu et al., 2017; Huo et al., 2019; Lv, 2020), including two intra- and three post-operative intervention studies. The heterogeneity is obvious, so we conducted a random-effects model, and performed subgroup analysis depending on the time points. The results of the meta-analysis revealed that the effect size of intraoperative intervention was RR = 2.07 (95% CI [1.09 to 3.95]). The effect size of postoperative intervention was slightly inferior, with RR = 1.14 (95% CI [1.01 to 1.22]). However, no significant difference was observed in the heterogeneity between intra- and post-operative groups (*p* = 0.066) **(** Figure 5B **)**.

#### Cardiac Function

**FIGURE 6:**
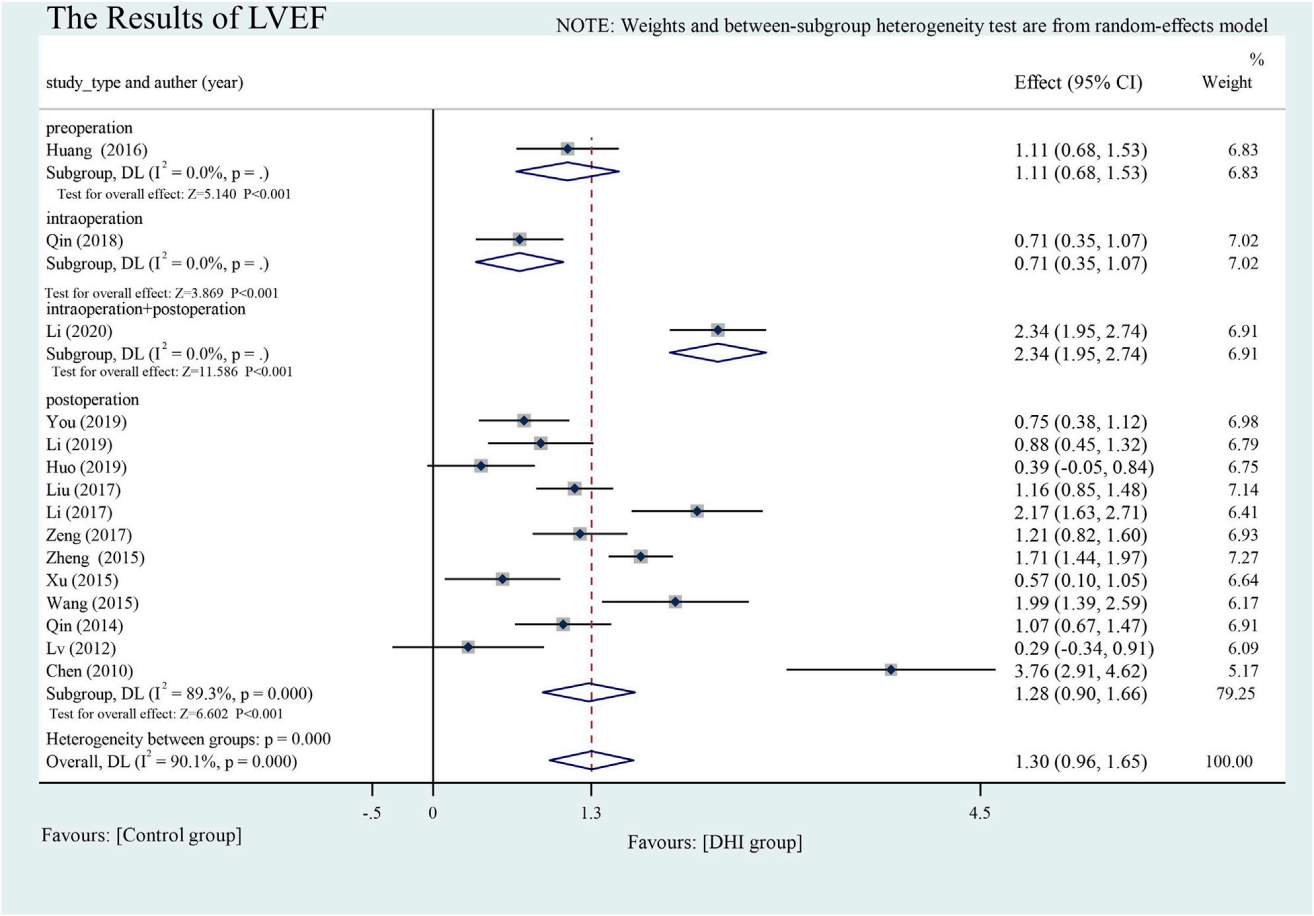
The results of LVEF.

The effects on LVEF were reported in one pre- (Huang, 2016), one intra- (Qin et al., 2018), one using both intra- and post- (Li and.Xu, 2020), and 12 post-operative studies. LVEF was significantly improved after DHI treatment compared with the control group (SMD = 1.30, 95% CI [0.96 to 1.65], *p* < 0.001). Significant heterogeneity was found between studies, so we used a random-effects model and conducted subgroup analysis. DHI administered at different time-points had significantly different effects on increasing LVEF: DHI administered intraoperatively and continued postoperatively was more effective in increasing LVEF than that other time-points (SMD = 2.34, 95% CI [1.95.2.74], heterogeneity between groups: *p* < 0.001). The source of heterogeneity was mainly from postoperative studies (I^2^fn2 = 89.3%, *p* < 0.001) **(** Figure 6 **)**.

#### Myocardial Injury Indices

The effect of DHI timing on myocardial injury indices could not be assessed, as only postoperative studies reported the effects on these indices.

**FIGURE 7:**
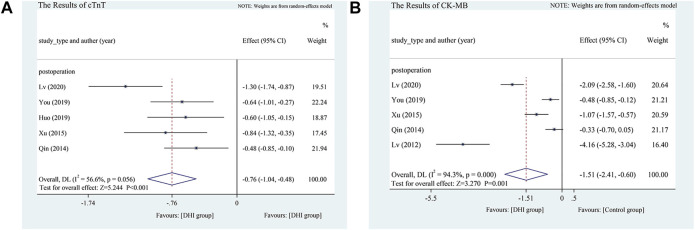
**(A)** The results of cTnT. **(B)** The results of CK-MB.

cTnT. Five postoperative intervention studies analyzed changes in cTnT levels (Qin et al., 2014; Xu et al., 2015; Huo et al., 2019; You, 2019; Lv, 2020). The random-effects model was used because heterogeneity between the groups was significant (*p* = 0.056, I^2^ = 56.6%). Compared with the control treatment, DHI could effectively reduce cTnT levels (SMD = −0.76, 95% CI [−1.04 to −0.48], *p* < 0.001) **(** Figure 7A **)**.

CK-MB. Five studies (all postoperative) analyzed changes in CK-MB levels (Lv, 2012; Qin et al., 2014; Xu et al., 2015; You, 2019; Lv, 2020). Because of the high heterogeneity (*p* < 0.1, I^2^ = 94.3%), the random-effects model was adopted. Compared with the control treatment, DHI was more effective in reducing CK-MB levels (SMD = −1.51, 95% CI [−2.41 to −0.60], *p* = 0.001) **(** Figure 7B **)**.

#### Indicators of Inflammatory Response

**FIGURE 8:**
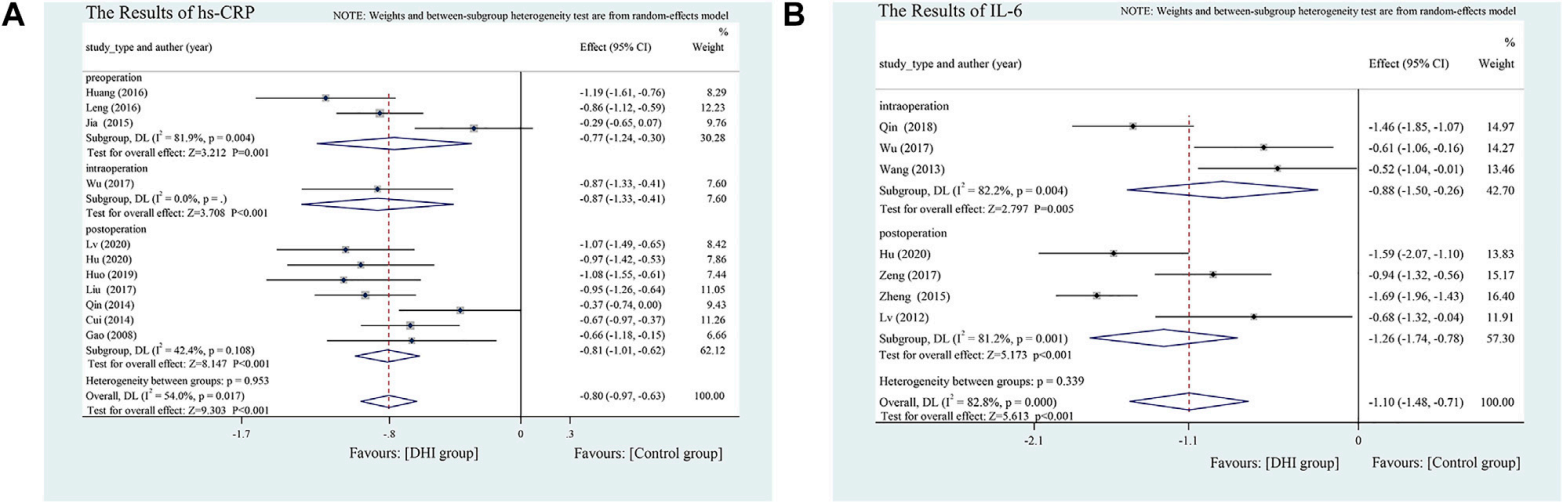
**(A)** The results of Hs-CRP. **(B)** The results of IL-6.

Hs-CRP. Eleven studies analyzed hs-CRP (Gao et al., 2008; Cui and Wang, 2014; Qin et al., 2014; Jia et al., 2015; Huang, 2016; Leng, 2016; Liu et al., 2017; Wu et al., 2017; Huo et al., 2019; Hu, 2020; Lv, 2020). There were significant differences between the two groups (SMD = −0.80, 95% CI [−0.97 to −0.63], *p* < 0.001) and because of significant heterogeneity, we adopted a random-effects model and performed a subgroup analysis according to the different DHI administration times during the perioperative period. Subgroup analysis showed that pre-, intra-, and post-operative DHI was more effective than conventional treatment, but there was no significant difference according to timing of DHI administration (*p* = 0.953). The heterogeneity of the postoperative intervention groups was significantly reduced after subgroup analysis (I^2^ = 42.4%, *p* = 0.108), while significant heterogeneity remained within the preoperative intervention groups (I^2^ = 81.9%, *p* = 0.004). This heterogeneity mainly resulted from the preoperative studies **(** Figure 8A **)**.

Serum IL-6. Seven studies analyzed serum IL-6 levels (Lv, 2012; Wang B. et al., 2013; Zheng et al., 2015; Wu et al., 2017; Zeng et al., 2017; Qin et al., 2018; Hu, 2020) and found significant differences between the DHI and control groups (SMD = −1.10, 95%CI [−1.48 to −0.71], *p* < 0.001). The test results showed significant heterogeneity (I^2^ = 82.8%, *p* < 0.1); hence, we divided them into an intra- and a postoperative PCI subgroup. In terms of the effect of DHI on IL-6 improvement, there was no significant difference between these subgroups (*p* = 0.339) **(** Figure 8B **)**.

### Subgroup Analysis of Postoperative Trials

The dose and course of DHI in the three preoperative studies were the same, with 20 ml delivered intravenously for 1 day. The three intraoperative intervention studies used the same intervention measures: 4 ml DHI injected intravenously at the beginning of the operation, and 20 ml intravenous infusion maintained until the end of the operation. However, the dose and duration of DHI were not uniform in the 16 studies after surgery, so we conducted a subgroup analysis for these studies.

The dosage of DHI after PCI varied from 20–40 ml, and the course of treatment spanned 7–14 days. There were no differences in MACEs, STR, LVEF, or cTnT between the different doses and durations. For hs-CRP, the effect of DHI at 14 days was better than that at 7 and 10 days (heterogeneity between the groups: *p* = 0.013), but there was no difference between different doses. For IL-6, a dosage of 30 ml proved most effective (heterogeneity between groups: *p* < 0.001), and there was no difference in efficacy between 10 and 14 days (*p* = 0.980). The 10-days treatment produced better results for CK-MB than for the other treatments (heterogeneity between groups: *p* < 0.001). **(** Table 3 **)**

**TABLE 3:** Subgroup analysis of postoperative trials.

Indices	Subgroups	Trials	Cases	RR [95%CI]	Z-value	*p*-value	Heterogeneity between groups
MACEs	40 ml	9	812	0.506 [0.402.0.637]	5.791	<0.001	NA
	7 d	2	231	0.741 [0.416.1.323]	1.013	0.311	*p* = 0.146
	14 d	7	581	0.464 [0.361.0.597]	5.976	<0.001	
STR	40 ml	4	310	1.144 [1.026.1.276 ]	2.422	0.015	NA
Indices	Subgroups	Trials	Cases	SMD [95%CI]	Z-value	*p*-value	Heterogeneity between groups
LVEF	20 ml	3	279	1.698 [0.174.3.222]	2.184	0.029	*p* = 0.759
	30 ml	2	390	1.314 [0.509.2.119]	3.200	0.001	
	40 ml	7	651	1.141 [0.696.1.586]	5.027	<0.001	
	7 d	3	321	0.894 [0.664.1.124]	7.621	<0.001	*p* = 0.155
	10 d	2	340	1.027 [−0.363.2.416]	1.448	0.148	
	14 d	7	659	1.546 [0.925.2.168]	4.875	<0.001	
Hs-CRP	20 ml	1	180	−0.947 [−1.255,−0.639]	6.020	<0.001	*p* = 0.688
	30 ml	2	266	−0.776 [−1.057,−0.496]	5.426	<0.001	
	40 ml	4	353	−0.785 [−1.149,−0.422]	4.233	<0.001	
	7 d	1	112	−0.369 [−0.742.0.005]	1.935	0.053	*p* = 0.013
	10 d	1	180	-0.673 [−0.973,−0.373]	4.390	<0.001	
	14 d	5	507	−0.959 [−1.143,−0.775]	10.204	<0.001	
IL-6	20 ml	1	40	−0.677 [−1.315,−0.039]	2.080	0.038	*p* < 0.001
	30 ml	2	386	−1.670 [−1.902,−1.438]	14.106	<0.001	
	40 ml	1	120	−0.941 [−1.318,−0.564]	4.886	<0.001	
	10 d	2	340	−1.229 [−2.223,−0.23]	2.424	0.015	*p* = 0.980
	14 d	2	206	−1.244 [−1.875,−0.614]	3.868	<0.001	
cTnT	40 ml	5	482	−0.760 [−1.044,−0.476]	5.244	<0.001	NA
	7 d	2	231	−0.560 [−0.824,−0.297]	4.173	<0.001	*p* = 0.157
	14 d	3	251	−0.918 [−1.337,−0.499]	4.294	<0.001	
CK-MB	40 ml	5	442	−1.506 [−2.408,−0.603]	3.270	0.01	NA
	7 d	2	231	−0.406 [−0.667,−0.146]	3.053	0.002	*p* < 0.001
	10 d	1	40	−4.161 [−5.284,−3.039]	7.268	<0.001	
	14 d	2	171	−1.581 [−2.580,−0.582]	3.103	0.002	

### Sensitivity Analysis and Meta Regression

We analyzed eight outcome indicators on forest plots as part of the meta-analysis. Marked heterogeneity was observed for all indicators, except for MACEs and reperfusion. Therefore, we re-conducted the meta-analysis after excluding the included studies a one-at-a-time. The heterogeneity of STR was changed from I^2^ = 72.8%, *p* = 0.002 to I^2^ = 30.8%, *p* = 0.216 when we excluded a single study of high risk bias (Wu et al., 2017). Furthermore, after the exclusion of one (Lv, 2020) of the five studies that analyzed cTnT, the results significantly changed from SMD = −0.76 (95% CI [−1.04 to −0.48], *p* < 0.0001; I^2^ = 56.6%) to SMD = −0.61 (95% CI [−0.82 to −0.41], *p* < 0.0001; I^2^ = 0%), indicating that the heterogeneity results of cTnT were unstable.

Eleven studies analyzed hs-CRP. Based on the analysis of pre-, intra-, and post-operative subgroups, we found that heterogeneity mainly arose from the preoperative studies (I^2^ = 81.9%, *p* = 0.004). After the exclusion of a single study (Jia et al., 2015), heterogeneity was significantly reduced (I^2^ = 39.7%, *p* = 0.198), indicating that the heterogeneity of hs-CRP was mainly derived from this report. This may have been related to the intraoperative use of nitro-glycerine in this study, as others only reported its use preoperatively.

**FIGURE 9:**
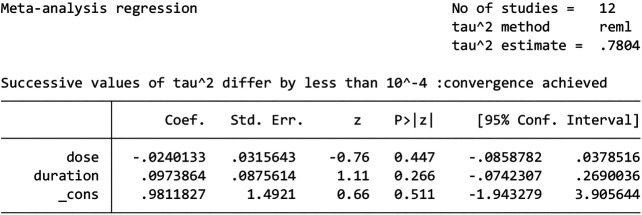
The results of meta-regression.

Compared to the previous results, CK-MB, IL-6 and LVEF values did not substantially change, and more than 10 studies assessed LVEF. As such, we conducted a meta-regression to further investigate the causes of heterogeneity in the 12 postoperative studies. We found that the baseline age and sex of the trial and control groups were consistent. Therefore, meta-regression was performed using two factors: dose and duration, but these were found not to be the sources of heterogeneity **(** Figure 9 **)**.

### Publication Bias

**FIGURE 10:**
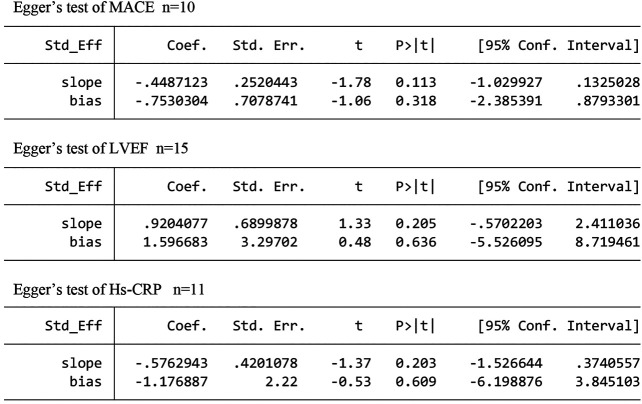
The publication bias analysis.

As more than 10 trials reported on MACEs, LVEF, and hs-CRP, we used an Egger’s test to objectively identify publication bias for these studies. The results showed respectable *p* values of 0.318, 0.636, 0.609, indicating an absence of publication bias **(** Figure 10 **)**.

## Discussion

### Overview

Twenty-three RCTs, including three pre-, three intra-, one both intra- and post-, and 16 post-operative treatment studies, were included in this systematic review to evaluate the efficacy of DHI during different time-points in the PCI perioperative period for AMI. Meta-analysis results showed that compared to control treatments, DHI was more effective at reducing cTnT, CK-MB, hs-CRP and IL-6 levels, and at improving LVEF and promoting reperfusion. This suggests that DHI can effectively reduce myocardial injury, reduce inflammation, improve cardiac function, promote vascular reflux, and reduce adverse cardiovascular events.

The intervention studies in which DHI was performed at different time points in the perioperative period uncovered distinctive profiles for the indicators assessed. Currently, the number of studies of pre- and intra-operative interventions is small, and these have mainly used inflammatory reactions, cardiac functions and reperfusion as observational indicators. The postoperative studies assessed inflammation, cardiac function, and reperfusion, and measured indicators of myocardial injury. Only time-points for reflow, LVEF and hs-CRP have been reported across pre-, intra-, and post-operative studies. Subgroup analysis showed that the best improvements in LVEF were seen when DHI was used at the beginning of surgery and continued after surgery rather than at a single time-point. In contrast, no significant differences were found for hs-CRP reductions and reflow improve when DHI was delivered at different time-points. Similarly, there was no statistical difference in MACEs, STR or IL-6 when DHI was used pre- or post-operatively. Finally, since cTnT and CK-MB are specific markers for the diagnosis of AMI, it was surprisingly that these factors were only measured in the postoperative studies. This is another area that should be addressed in future studies, whereby cTnT and CK-MB should be assessed pre- and intra-operatively.

We performed a subgroup analysis of 16 postoperative studies based on different doses and duration, which showed that the greatest IL-6 inhibition was achieved with a 30 ml dose of DHI compared to that of 20 ml or 40 ml. For hs-CRP, treatment over a 14 days postoperative period was more effective than that of other treatment courses. However, for CK-MB levels, the best results were seen with a 10-days postoperative treatment period. However, the evidence for these findings comes from small samples, reducing their strength. We expect more clinical trials with large samples to explore these issues.

In addition, we noted that starting DHI during surgery can improve cardiac function, and also reduce the required postoperative dose (4 ml per day for 14 days), which will help to reduce medical costs. However, this evidence only comes from a single study (Li and Xu, 2020), and its conclusion is open to debate.

### PCI Core Indicators and the Mechanism of Acton of DHI

The incidence of MACEs after PCI is a primary concern, and reperfusion is an independent predictor of death and myocardial infarction after percutaneous coronary intervention (Resnic et al., 2003). For these reasons, we used MACEs and reperfusion as the major indicators in our study. In the early stage of myocardial ischemia reperfusion, inflammatory factors such as IL-6 and hs-CRP activate white blood cells, causing them to adhere to the microvascular endothelial cells in the ischemic area, resulting in endothelial cell atrophy, rupture, and finally necrosis (Jong et al., 2016). These necrotic endothelial cells can enter the myocardial ischemic area, producing more cytokines, which will eventually further aggravate the myocardial tissue injury (Wang et al., 2012). The salvianolic acid B component of DHI acts to inhibit apoptosis in myocardial cells by reducing the expression of the Bax signal transduction and transcriptional activation factor, and suppressing the expression of IL-6 (Chen et al., 2018), thereby reducing the myocardial tissue injury. In the six pre- and intra-PCI studies, it was also suggested that DHI may stimulate reflux through its anti-inflammatory effects (Jia et al., 2015; Wu et al., 2017). Our meta-analysis showed that the use of DHI before and after PCI was more effective in promoting reflux than the use of DHI after PCI, and this result may be related to the anti-inflammatory effect of DHI in the early stages of myocardial ischemia.

Both CK-MB and cTnT are used as the “gold standard” for AMI diagnosis, while LVEF is a powerful prognostic predictor in patients with CAD (Allman et al., 2002). Subtle increases in CK-MB and cTnT levels caused by PCI are sufficient to significantly increase the long-term risk of death (Brener et al., 2002; Ioannidis et al., 2003). The tanshinone and safflower yellow components of DHI can improve energy metabolism and promote the scavenging of oxygen free radicals to improve myocardial ischemia-reperfusion injury (Zhang et al., 2009). Our meta-analysis results suggested that postoperative DHI can effectively reduce CK-MB and cTnT levels, and early application of DHI at the beginning of surgery and continued application after surgery can improve cardiac function more effectively.

AMI occurs when blood flow in the coronary artery is blocked and the heart muscle is continuously ischemic, PCI is one of the first-line therapeutic strategies for acute AMI, and its purpose is to restore coronary blood flow (Santoro et al., 2008). The conclusion of our study showed that DHI can effectively improve perioperative reperfusion of PCI, reduce myocardial injury and inflammation, improve cardiac function and reduce the incidence of adverse cardiovascular events. In the China Medicine Information Platform (https://www.dayi.org.cn/), DHI is classified as nourishing blood and promoting blood circulation agent, and its effect is “promoting blood circulation, removing blood stasis, expanding arteries and relieving collaterals”. From the point of view of modern medicine, DHI is more likely to be an anti-platelet agglutination drug and vasodilator drug. A large number of studies have confirmed that the active components in Salvia miltiorrhiza Bunge, such as salviol, danshensu and protocatechuic aldehydes, can activate prostaglandin G/H synthase two and promote the synthesis of prostaglandin (Zhu et al., 2019). The main effect of prostaglandin is to prevent platelet aggregation and depolymerize platelet emboli (Hulshof et al., 2020), which may explain the “promoting blood circulation and removing blood stasis” of DHI. At the same time, prostaglandins have vasodilating effects (Wang D. et al., 2013), in addition, DHI may also act as a vasodilator by inhibiting voltage-dependent Ca^2+^ release and inositol 3-phosphate (IP3) receptor-mediated Ca^2+^ influx (Zhi et al., 2012).

### Comparison With Previous Systematic Reviews

The effect of DHI in the perioperative period was previously assessed in a systematic review by Zhang et al., who concluded that DHI may improve reperfusion after PCI (Zhang et al., 2020). After a meta-analysis of 1,131 patients in 12 studies, Yuan et al. concluded that DHI can improve the perioperative cardiac function in patients with AMI and increase the TIMI blood flow grade (Yuan et al., 2020). Shi et al. compared the effects of eight types of traditional Chinese patent medicine injections, including DHI, on promoting blood circulation and removing blood stasis by recording effectivity rates, MACE incident rates, and LVEF (Shi et al., 2019). Yang et al. showed that DHI could have a protective role in the myocardium by reducing the levels of inflammatory factors (hs-CRP, IL-6, and matrix metalloprotein-9) after coronary intervention and improving myocardial injury indicators (CK-MB and cTnT) (Yang et al., 2018). From the perspective of endothelial function, He et al. systematically demonstrated that DHI can improve endothelial function after PCI for coronary heart disease (He et al., 2018). The systematic review of DHI performed by Zhang et al. also concluded that it could improve the degree of myocardial necrosis, inhibit the inflammatory response, reduce the oxidative stress and endothelial function injury, and reduce the incidence of cardiovascular events after PCI (Zhang et al., 2017). Thus, DHI appears to protect the myocardium during the perioperative period of PCI. However, none of these studies evaluated the efficacy of DHI at different time-points across the perioperative period.

Our meta-analysis results showed that compared to control treatments, DHI was more effective at reducing cTnT, CK-MB, hs-CRP, and IL-6 levels, improving LVEF, and promoting reperfusion, which is consistent with the previous reviews. In contrast to these studies, however, we focused our analysis on the efficiency of DHI before, during, and after PCI. Subgroup analysis suggested that early use of DHI in the perioperative period was superior to use of DHI after PCI in terms of improving cardiac function. In postoperative intervention, there was no difference between different doses and courses of DHI for MACE, LVEF, and cTnT. However, IL-6 inhibition was achieved with a 30 ml dose of DHI compared to that of 20 ml or 40 ML, for hs-CRP, treatment over a 14 day period was more effective than that of other treatment courses, for CK-MB levels, the best results were seen with a 10-days treatment period. Additional systematic reviews focusing on the efficacy of DHI at different time points during perioperative period are required to confirm these findings.

### Limitations and Implications for Further Research

There are limitations to our meta-analysis. First, due to the limited number of original studies, particularly in terms of pre- and intra-operative interventions in PCI, conclusions could only be drawn in regard to reflow, cardiac function, and inflammation levels, rather than in the quantitative analysis of myocardial injury outcomes. When more RCTs are available in the literature, we will update the systematic review. Second, all included RCTs were linked to a high risk of bias, RCTs related to DHI in the treatment of AMI should be reported following the CONSORT guidelines in order to improve the quality of the trials. Third, the reports used in the systematic review were limited to China and the sample size in the study was very small, further multinational research is needed. Forth, the diagnostic and curative effect criteria were inconsistent across studies, with some reports adopting the traditional Chinese medicine-based diagnostic criteria and others using Western medicine-based diagnostic criteria. This may limit the inferences made by our study. Fifth, some results of the meta-analysis showed large heterogeneity, which was not be eliminated by sensitivity analysis or meta-regression. Baseline conditions of patients, such as underlying disease, infarct site, and BMI, were not reported in some studies, and these factors may influence the final outcomes and heterogeneity. Therefore, we intend to explore the factors that affect prognosis of patients with AMI in our future research. Interestingly, we note that there is a multi-centre, prospective, randomized, evaluator-blind study initiated in 2020 by Zhang et al. (Zhang et al., 2020). This represents the first and largest well-designed, multi-centre randomized controlled trial with rigorous quality control to evaluate the efficacy and safety of DHI, as well as to determine the optimal timing of delivery to prevent MACE in patients with ST-elevation myocardial infarction. We hope that additional large, well-designed, multi-centre, randomized, and placebo-controlled double-blind trials will be conducted to verify and explore the best time to administer DHI.

## Conclusion

DHI is effective at all stages of perioperative PCI for AMI. The best improvements in LVEF were seen when DHI was used at the beginning of surgery and continued after surgery rather than at a single time-point. After the operation, 30 ml is recommended to inhibit IL-6 levels, for patients with high hs-CRP, a course of 14 days is recommended, for patients with obvious abnormalities of CK-MB, a 10-days course of treatment is recommended. However, the small sample size of the assessed studies means that their quality is not high. Thus, large, multi-centre RCTs are needed in order to validation of our findings.

## Data Availability

Publicly available datasets were analyzed in this study. The datasets used and/or analyzed during the current study are available from the corresponding author on reasonable request.
