# Supplementary material for: Comparison of the Efficacy of Danhong Injections at Different Time-points During the Perioperative Period of Acute Myocardial Infarction: A Systematic Review and Meta-analysis of Randomized Controlled Trials
Source: Front Pharmacol. 2021 Apr 29;12:643446. doi: 10.3389/fphar.2021.643446 (PMC8117241; doi:10.3389/fphar.2021.643446)
Supplement: Supplementary file 1 [file datasheet1.docx]

Supplementary Material

# Supplementary data sheet 1

## The product information of Danhong injection


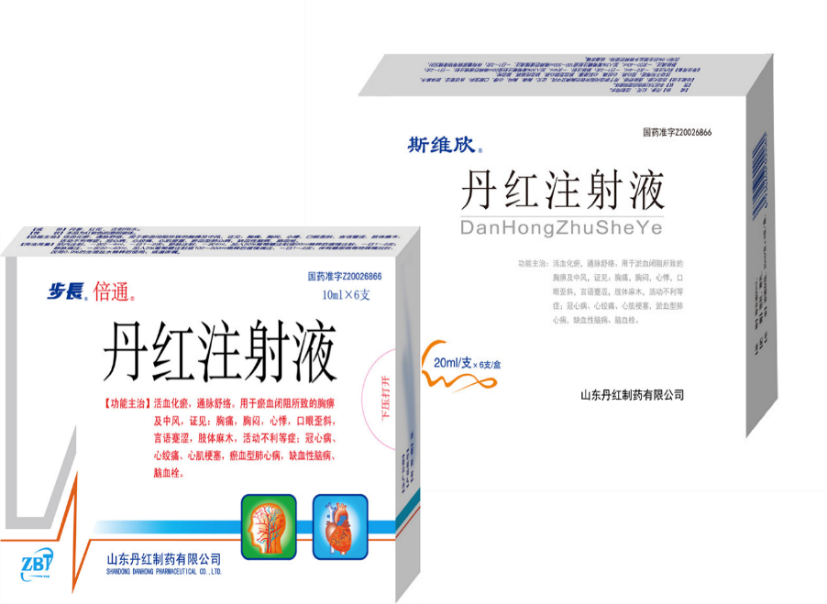
About the composition of the DHI and how these were reported in the original studies, all produced by Shandong Danhong Pharmaceutical Co., Ltd. Therefore, we listed them as one row.

Figure S1 Danhong Injection

In the China Medicine Information Platform (https://www.dayi.org.cn/), DHI is classified as nourishing blood and promoting blood circulation agent.

From the point of view of modern medicine, DHI is more likely to be an anti-platelet agglutination drug and vasodilator drug.


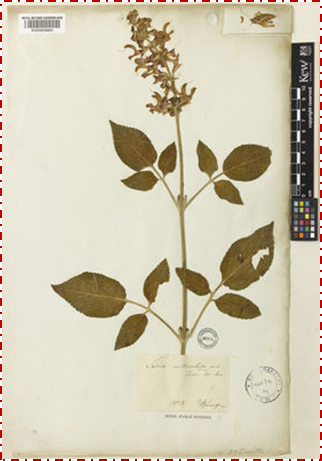
Figure S2 The Image of Salvia miltiorrhiza Bunge[Lamiaceae]

(The image is from Plants of the World Online http://plantsoftheworldonline.org/taxon/urn:lsid:ipni.org:names:456707-1)


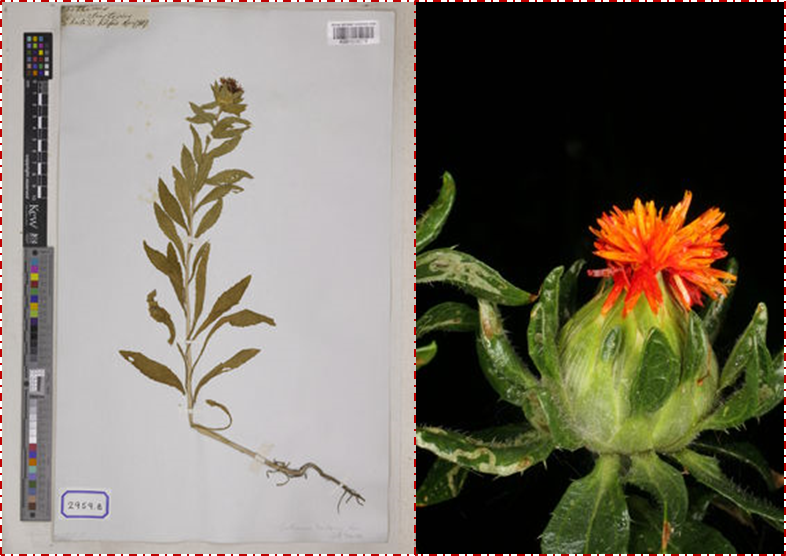


Figure S3 The Image of Carthamus tinctorius L.[Asteraceae]

(The image is from Plants of the World Online <http://plantsoftheworldonline.org/taxon/urn:lsid:ipni.org:names:324467-2>)

## More details about the product information of Danhong injection

| **Name** | **Source** | **Species**  **Raw materials** | **Chemical composition criteria** | **Phytochemical compositions** | **Therapeutic claims in TCM** | **Indications** | **Adverse drug reactions** | **Quality control reported? (Y/N)** | **Chemical analysis reported? (Y/N)** |
| --- | --- | --- | --- | --- | --- | --- | --- | --- | --- |
| Danhong injection | Shandong Danhong Pharmaceutical Co., Ltd.  National medicine approval Z20026866 | For each 1000mL DHI,  Salvia miltiorrhiza Bunge, 750g,  Carthamus tinctorius L., 250g,  sodium chloride for injection 7g. | Tanshinol,  >0.5mg/1mL; Protocatechualdehyde,  >0.5mg/1mL;  General flavone,  5mg/1mL. | Tanshinol,  Dan phenolic acids D,  Protocatechualdehyde，  General flavone，  P-coumaric acid, etc. | Promoting blood circulation to remove blood stasis and dredging meridians | Coronary heart disease, angina pectoris, myocardial infarction, cor pulmonale of stasis type, ischemic encephalopathy, cerebral thrombosis, etc. | 1. Allergic reactions: facial flushing, rash, pruritus, urticaria, laryngeal edema, dyspnea, suffocation, palpitation, cyanosis, decreased blood pressure, anaphylactic shock, etc.  2. Systemic damage: chills, high fever, etc.  3. Cardiovascular system damage: chest tightness, palpitation, elevated blood pressure, etc.  4. Digestive system damage: nausea, vomiting, abdominal pain, diarrhea, etc., abnormal cases of liver biochemical indicators have been reported.  5.nervous system damage: dizziness, headache, convulsions, coma, etc.  6. Other cases: purpura, hematuria, epistaxis, gingival bleeding, conjunctival bleeding, gastrointestinal bleeding, subcutaneous bleeding points and ecchymosis were reported. | Y-National Food and Drug Administration National Drug Standards, WS-11220(ZD-1220)-2002-2017Z | N |

1. **The chemical characterisation of Danhong injection**

The information comes from the National Medical Products Administration (available at: <https://www.nmpa.gov.cn/>) and Drug Standards Database (available at: <https://www.drugfuture.com/standard/>).

**[Approval number]** National medicine approval Z20026866

**[The product name]** Danhong injection

**[Prescription]** Salvia miltiorrhiza Bunge 750g Carthamus tinctorius L. 250g sodium chloride for injection was prepared into 1000mL

**[Method]** The above two medicinal materials, Salvia miltiorrhiza Bunge with dilute ethanol warm soak twice, 1 hour each time, filtration, filtrate reserve. The residue was mixed with Carthamus tinctorius L., soaked with water for two times, 1 hour each time, filtered, filtrate was combined, concentrated to 1.10-1.20 (65℃) ointment, sodium chloride for injection was added to isosmotic, PH value was adjusted to 6-7, filtered, refrigerated for 24 hours, water for injection was added to the specified amount, filtered, encapsulation, sterilized.

**[Properties]** This product is a reddish-brown clear liquid.

**[Identification]** Take 4 mL of the product, dry it, add anhydrous ethanol 1 mL to the residue, dissolve it, place it, centrifuge it, take the supernatant as the test product solution. In addition, 1g of Carthamus tinctorius L. control medicine was taken, and 10 mL of water was added for ultrasonic treatment for 30 minutes. After filtration, the filtrate was concentrated to dry, the residue was added with anhydrous ethanol 1 mL, dissolved, placed and centrifuged, and the supernatant was taken as the control medicine solution. According to the thin layer chromatography (Chinese pharmacopoeia 2000 edition of an appendix VIB) test, absorb the above two solutions each 1 μL, respectively point on the same silica gel G thin layer plate, with n-butanol-acetic acid monowater (6:2.4:5) as the development agent, expand, take out, dry, under the ultraviolet lamp (365nm) inspection. Fluorescent spots of the same color were found in the chromatographic position of the test material and the control material.

**[Check]**

**PH value** Should be 4.5~6.5 (Appendix VII G, Chinese Pharmacopoeia 2000 edition). Take 1 mL of this product, add 1~3 drops of tannic acid test solution, and avoid turbidity.

**On ignition residue** Take 10mL of this product and check it according to law (Appendix IXJ, 2000 edition of Chinese Pharmacopoeia, should not exceed 1.5% (g/mL).

**The hemolysis test** 2% of red cell suspension liquid preparation: take the rabbit heart blood, with glass beads of the container, the vibration wave for 10 minutes, remove the fibrinogen, made into fine blood, with physiological sodium chloride solution, shake well, centrifugal, leaning to the supernatant, precipitation of red blood cells with physiological sodium chloride solution washing 3~4 times, until after centrifugal supernatant do not show red, the red blood cell volume with physiological sodium chloride solution diluted to 2% of the mixed suspension, quick. Use the same day and shake well when used.

**Test method** In test tube 5 pieces, serial number, 1~3 pipe add 0.3 mL of the test and normal saline respectively 2.2 mL, 4 tube join physiological sodium chloride solution of 2.5 mL (for a negative charge), 5 tube join 2.5 mL distilled water (for a positive charge) added 2% to each and red cell suspension liquid 2.5 mL, constant temperature box, keep the temperature of 36.5±0.5℃, observe 3 hours, should be no hemolysis phenomenon.

**Pyrogen** Take this product and check it according to law (Appendix XIIIA of Chinese Pharmacopoeia 2000 edition). The dose should be injected into 2mL per 1kg of rabbit body weight, which should meet the regulations.

**Others** All relevant requirements under the testing method for injections and injection-related substances shall be complied with (Appendix I U and IXS of Chinese Pharmacopoeia 2000 Edition).

**[Content determination]**

**Salvia miltiorrhiza Bunge** The results were determined by HPLC (Chinese Pharmacopoeia 2000 edition Appendix VID).

Chromatographic conditions and system suitability test Octadecyl silane bonded silica gel was used as filler. Methanol-1% glacial acetic acid solution (13:87) was used as mobile phase: the detection wavelength was 280nm.The theoretical plate number should not be less than 5000 according to Danshensu peak.

Preparation of reference solution An appropriate amount of Danshensu Sodium and protocatechualdehyde was accurately weighed and added with water respectively to make a solution containing 5μg each per 1mL to obtain.

Preparation of test solution Precise measurement: Take 5mLof this product, put it into a 20mL flask, dilute it with water to the scale, shake it evenly, and get it.

Measurements Precise absorption of reference and test solution 10ul respectively, injection liquid chromatograph, determination, is obtained.

Each LML of this product contains the total amount of Salvia miltiorrhiza Bunge, Danshensu (C9H10O5) and protocatechualdehyde (C7H6O3), not less than 0.5mg.

**General flavone**

Preparation of reference solution Take 20mg of reference rutin dried at 120℃ to constant weight, put it into a 100mL flask, add an appropriate amount of 50% methanol, shake it to dissolve and dilute it to the scale, shake it evenly, get (each 1mL contains 0.2mg of anhydrous rutin).

Preparation of standard curves Precisely take 1.0mL, 2.0mL, 3.0mL, 4.0mL and 5.0mL of reference solution, put them into 10mL flask respectively, add 50% methanol to 5mL each, add 0.3mL of 5% sodium nitrite solution, shake them well, leave them for 6 minutes, shake them well, add 0.3mL of 10% aluminum nitrate solution, shake them well, leave them for 6 minutes, add 4mL of sodium hydroxide test solution, add 50% methanol to scale, shake them well. Leave the corresponding solution blank. Spectrophotometry (Appendix V B of Chinese Pharmacopoeia 2000 edition), the absorbance was measured at the wavelength of 500nm, and the standard curve was drawn with absorbance as the ordinate and concentration as the abscissa.

Measurements Precisely absorb 5mL of this product, put it in a 100mL flask, dilute it with water to scale, and shake it well. Precisely take 1mL, put it in a 10mL flask, add 50% methanol to scale, shake it well, and use it as blank control. In addition, a precise quantity of 1mL was taken and put into a 10mL flask. According to the method under the preparation of standard curve, the absorbance was immediately measured according to law starting from "adding 50% methanol to 5mL". The weight of rutin in the test solution was read out from the standard curve and calculated.

Rutin (C27H30O16) should not be less than 5.0mg of total flavonoids per 1mL of this product.

**[Function]**

Activate blood circulation, remove blood stasis, pass arteries and relax luo. For chest pain caused by blood stasis and stroke, the syndrome can be seen as: chest pain, chest tightness, palpitation, mouth and eye askew, language Jian astringent, numbness of limbs, adverse activities, etc. Coronary heart disease, angina pectoris, myocardial infarction, ischemic encephalopathy, cerebral thrombosis and stasis of cor pulmonale.

**[Usage and Dosage]**

Intramuscular injection, 2~4mL once, 1~2 times a day; intravenous injection, 4mL at a time, adding 50% glucose injection 20mL after dilution, slowly injected, 1~2 times a day; intravenous infusion, 20~60mL once, adding 5% Glucose injection 100~500mL diluted and slowly dripping, 1~2 times a day; or follow a doctor's advice.

**[Taboo]**

Not to be used in pregnant women.

**[Notice]**

(1) This product should not be mixed with other drugs in the same container for use;

(2) This product is pure traditional Chinese medicine preparation, improper preservation may affect the quality of the product. It can not be used when the liquid reappears turbidity, precipitation, discoloration, air leakage and other phenomena.

**[Specification]**

Each (1) 2mL (2)10mL (3) 20mL

**[Storage]**

Sealed and protected from light.

**[Validity]**

1.5 years.
